# Influence of Driving and Transportation Access on Social Isolation Risk Among Older Adults

**DOI:** 10.1093/geroni/igaa057.959

**Published:** 2020-12-16

**Authors:** Matthew Smith, Caroline Bergeron, Matthew Barrett, Leigh Ann Eagle, Sue Lachenmayr

**Affiliations:** 1 Texas A&M University, College Station, Texas, United States; 2 Institut national de santé publique du Québec, Québec City, Quebec, Canada; 3 MAC, Salisbury, Maryland, United States

## Abstract

Background. Transportation is essential to accessing healthcare and community services, but the inability to find transportation may hinder social interactions and connectivity. This study examined driving and transportation access associated with self-reported social isolation risk among adults age 60 years and older. Methods. The Upstream Social Isolation Risk Screener (U-SIRS) was developed to assess social isolation risk among older adults within clinical and community settings. Comprised of 13 items (Cronbach’s alpha=0.80), the U-SIRS assesses physical, emotional, and social support aspects of social isolation. Using an internet-delivered survey, data were analyzed from a national sample of 4,082 adults age 60 years and older. Theta scores for the U-SIRS served as the dependent variable, which were generated using Item Response Theory. An ordinary least squares regression model was fitted to identify transportation-related indicators associated with social isolation risk. Results. Approximately 13% of participants did not drive and 18.2% reported not being able to identify a ride or transportation when needed. Higher U-SIRS scores were reported among participants who did not drive (B=0.034, P=0.020). Lower U-SIRS scores were reported among those who live with a spouse/partner (B=-0.153, P<0.001) and those who reported the ability to get a ride from a family member (B=-0.160, P<0.001), friend (B=-0.256, P<0.001), or taxi (B=-0.032, P=0.044). Every additional source of transportation available significantly reduced participants’ U-SIRS score (B=-0.239, P<0.001). Conclusion. Given transportation options may reflect physical functioning, social networks, and socioeconomic status, study findings suggest transportation access is an important contextual factor associated with social isolation risk.

